# Rabies post-exposure prophylaxis started during or after travel: A GeoSentinel analysis

**DOI:** 10.1371/journal.pntd.0006951

**Published:** 2018-11-13

**Authors:** Philippe Gautret, Kristina M. Angelo, Hilmir Asgeirsson, David G. Lalloo, Marc Shaw, Eli Schwartz, Michael Libman, Kevin C. Kain, Watcharapong Piyaphanee, Holly Murphy, Karin Leder, Jean Vincelette, Mogens Jensenius, Jesse Waggoner, Daniel Leung, Sarah Borwein, Lucille Blumberg, Patricia Schlagenhauf, Elizabeth D. Barnett, Davidson H. Hamer, GeoSentinel Global Surveillance Network

**Affiliations:** 1 Institut Méditerranée Infection, Aix-Marseille University, Marseille, France; 2 Division of Global Migration and Quarantine, Centers for Disease Control and Prevention, Atlanta, Georgia, United States of America; 3 Department of Infectious Diseases, Karolinska University Hospital, and Unit of Infectious Diseases, Department of Medicine Huddinge, Karolinska Institutet, Stockholm, Sweden; 4 Department of Clinical Sciences, Liverpool School of Tropical Medicine, Liverpool, United Kingdom; 5 Public Health and Tropical Medicine Department, James Cook University, Townsville, Australia, and WORLDWISE Travellers Health Centres of New Zealand; 6 The Center of Geographical Medicine, Sheba Medical Center, Tel HaShomer, and Sackler Faculty of Medicine, Tel Aviv University, Tel Aviv, Israel; 7 J.D. MacLean Centre for Tropical Diseases, McGill University, Montreal, Canada; 8 Tropical Disease Unit, UHN-Toronto General Hospital, University of Toronto, Toronto, Canada; 9 Department of Clinical Tropical Medicine, Faculty of Tropical Medicine, Mahidol University, Bangkok, Thailand; 10 CIWEC Hospital and Travel Medicine Center, Kathmandu, Nepal; 11 School of Epidemiology and Preventive Medicine, Monash University, and Victorian Infectious Disease Service, Royal Melbourne Hospital at the Doherty Institute for Infection and Immunity, Melbourne, Australia; 12 Clinique Santé-voyage, Fondation du CHUM, Université de Montréal, Montreal, Canada; 13 Department of Infectious Diseases - Oslo University Hospital, Oslo, Norway; 14 Department of Medicine, Division of Infectious Diseases, Emory University School of Medicine, Atlanta, Georgia, United States of America; 15 Division of Infectious Diseases, University of Utah School of Medicine, Salt Lake City, Utah, United States of America; 16 TravelSafe Medical Centre - Central Health Medical Practice, Hong Kong, China; 17 National Institute for Communicable Diseases, South Africa; 18 University of Zürich Centre for Travel Medicine, WHO Collaborating Centre for Travellers’ Health, Epidemiology, Biostatistics and Prevention Institute, University of Zürich, Switzerland; 19 Maxwell Finland Laboratory for Infectious Diseases, Boston Medical Center, and Department of Pediatrics, Boston University School of Medicine, Boston, Massachusetts, United States of America; 20 Department of Global Health, Boston University School of Public Health and Section of Infectious Diseases, Department of Medicine, Boston University School of Medicine, Boston, Massachusetts, United States of America; US Department of Agriculture, UNITED STATES

## Abstract

**Background:**

Recent studies demonstrate that rabies post-exposure prophylaxis (RPEP) in international travelers is suboptimal, with only 5–20% of travelers receiving rabies immune globulin (RIG) in the country of exposure when indicated. We hypothesized that travelers may not be receiving RIG appropriately, and practices may vary between countries. We aim to describe the characteristics of travelers who received RIG and/or RPEP during travel.

**Methodology/Principal findings:**

We conducted a multi-center review of international travelers exposed to potentially rabid animals, collecting information on RPEP administration. Travelers who started RPEP before (Group A) and at (Group B) presentation to a GeoSentinel clinic during September 2014–July 2017 were included. We included 920 travelers who started RPEP. About two-thirds of Group A travelers with an indication for rabies immunoglobulin (RIG) did not receive it. Travelers exposed in Indonesia were less likely to receive RIG in the country of exposure (relative risk: 0.30; 95% confidence interval: 0.12–0.73; P = 0.01). Travelers exposed in Thailand [Relative risk (RR) 1.38, 95% Confidence Interval (95% CI): 1.0–1.8; P = 0.02], Sri Lanka (RR 3.99, 95% CI: 3.99–11.9; P = 0.013), and the Philippines (RR 19.95, 95% CI: 2.5–157.2; P = 0.01), were more likely to receive RIG in the country of exposure.

**Conclusions/Significance:**

This analysis highlights gaps in early delivery of RIG to travelers and identifies specific countries where travelers may be more or less likely to receive RIG. More detailed country-level information helps inform risk education of international travelers regarding appropriate rabies prevention.

## Author summary

International travelers may be exposed to rabid animals while traveling abroad. Current guidelines recommended that any traveler who was not been vaccinated against rabies before travel and sustained an animal exposure putting him at risk for rabies required rabies post-exposure prophylaxis including rabies immunoglobulin administration in addition to rabies vaccine as soon as possible. Available data indicate that only a small proportion of travelers received rabies immunoglobulin in the country of exposure when indicated. In this multi-center survey, we collected information on rabies post-exposure prophylaxis administration in international travelers exposed to potentially rabid animals. We observed that about two-thirds of travelers with an indication for rabies immunoglobulin who started their post-exposure prophylaxis during travel did not receive it. This analysis identified specific countries where travelers may be more or less likely to receive rabies immunoglobulin and helps inform risk education of international travelers regarding appropriate rabies prevention. In our analysis, Bali, Indonesia, was the most common location to have an exposure to a potentially rabid animal, but very few travelers received rabies immunoglobulin in Indonesia when indicated. By contrast, travelers exposed in Thailand, Sri Lanka and the Philippines were more likely to receive rabies immunoglobulin in the country of exposure.

## Introduction

International travelers may be exposed to rabid animals while traveling abroad. The estimated incidence of potential rabies exposures requiring post-exposure prophylaxis (RPEP) among international travelers is 0.4 per 1,000 per month of stay [1]. The proportion of international travelers requiring RPEP at GeoSentinel clinics among other patients increased from <0.5% of visits in 2003 to >2% in 2012. The increase may be due to greater diversity of travel destinations and number of international travelers [2].

Limited data are available on the proportion of international travelers that receive pre-travel rabies vaccination, but vaccine provision is usually guided by individual risk assessment and cost [1]. At the time of writing, World Health Organization (WHO) guidelines recommended that any traveler who had not received a three-dose pre-exposure prophylaxis (PrEP) and sustained a category III exposure—transdermal bite(s) or scratch(es), licks to broken skin, mucus membrane contamination, or contact with a bat—required rabies immunoglobulin (RIG) administration in addition to rabies vaccine [3]. Pre-exposure immunization obviates the need for RIG after exposure [3]. Recent studies demonstrate that only 5–20% of travelers received RIG in the country of exposure when indicated [4–12] (Table 1).

**Table 1:** Summary of studies showing proportion of patients with indication for rabies immunoglobulin who received rabies immunoglobulin in the country of exposure.

Year	Number of patients	Study design	Proportion of patients with indication for RIG who received RIG in the country of exposure^2^	Reference
1997–2005	261	Multicenter (France, Australia, New Zealand)	11.2%	[4]
2000–2009	139	Unicenter (United Kingdom)	3.8%	[5]
1998–2012	363	Unicenter (New Zealand)	20.3%	[6]
2000–2012	1,126	Unicenter (Denmark)	10.3%	[7]
2008–2010	45	Multicenter (France, Australia, New Zealand, Singapore) ^1^	5.3%	[8]
2006–2012	106	Unicenter (Republic of Korea)	9.4%	[9]
2007–2011	780	Unicenter (Australia)	9.0%	[10]
2008–2012	136	Unicenter (Australia)	4.0%	[11]
2009–2010	65	Unicenter (Australia)	7.8%	[12]

^1^Patients exposed in Bali, only

^2^According to the WHO criteria (category III exposure in patients who received no, one, or two doses of rabies vaccine, before travel) [3]

WHO = World Health Organization, RIG = rabies immunoglobulin

The objective of this analysis was to conduct a multi-center review of international travelers exposed to potentially rabid animals. We describe the characteristics of travelers who received RIG and/or RPEP during travel in the region of exposure, to determine whether receipt of RIG varied by country or region.

## Methods

### Data source

GeoSentinel is a global clinician-based sentinel surveillance system that monitors travel-related illness and other conditions among international travelers. It was established in 1995 as a collaboration between the Centers for Disease Control and Prevention (CDC) and the International Society of Travel Medicine. GeoSentinel currently consists of 70 specialized travel and tropical medicine clinical sites in 31 countries [13]. GeoSentinel’s data collection protocol has been reviewed by the CDC’s National Center for Emerging and Zoonotic Infectious Diseases and is classified as public health surveillance and not human subjects research. Additional ethics clearance was obtained by participating sites, as required by their respective institutions.

### Survey of GeoSentinel travelers with a potential rabies exposure

We analyzed records submitted to GeoSentinel of international travelers with exposure to a potentially rabid animal. Demographics and travel history, place of exposure, and animal involved in the exposure were recorded. Travelers who started RPEP before (Group A) and at (Group B) presentation to a GeoSentinel clinic during September 2014–July 2017 were included. Among travelers in the former group, data were collected and assessed for type of exposure [3], rabies PrEP, occurrences where RPEP with or without RIG should have been administered, and differences between international travelers who received RIG in the country of exposure and those who did not (relative risk). We excluded records if they were not travel-related or the exposure country was unascertainable.

### Statistical analysis

Data were managed using Microsoft Access (Redmond, Washington, USA). Demographic analyses were descriptive; for the binary outcome variable of receiving RIG in the country of exposure, relative risks (RR) and 95% confidence intervals (95% CI) were determined. Statistical significance was defined as P<0.05. All analyses were performed using SAS version 9.4 (Cary, NC, USA).

## Results

**Fig 1:**
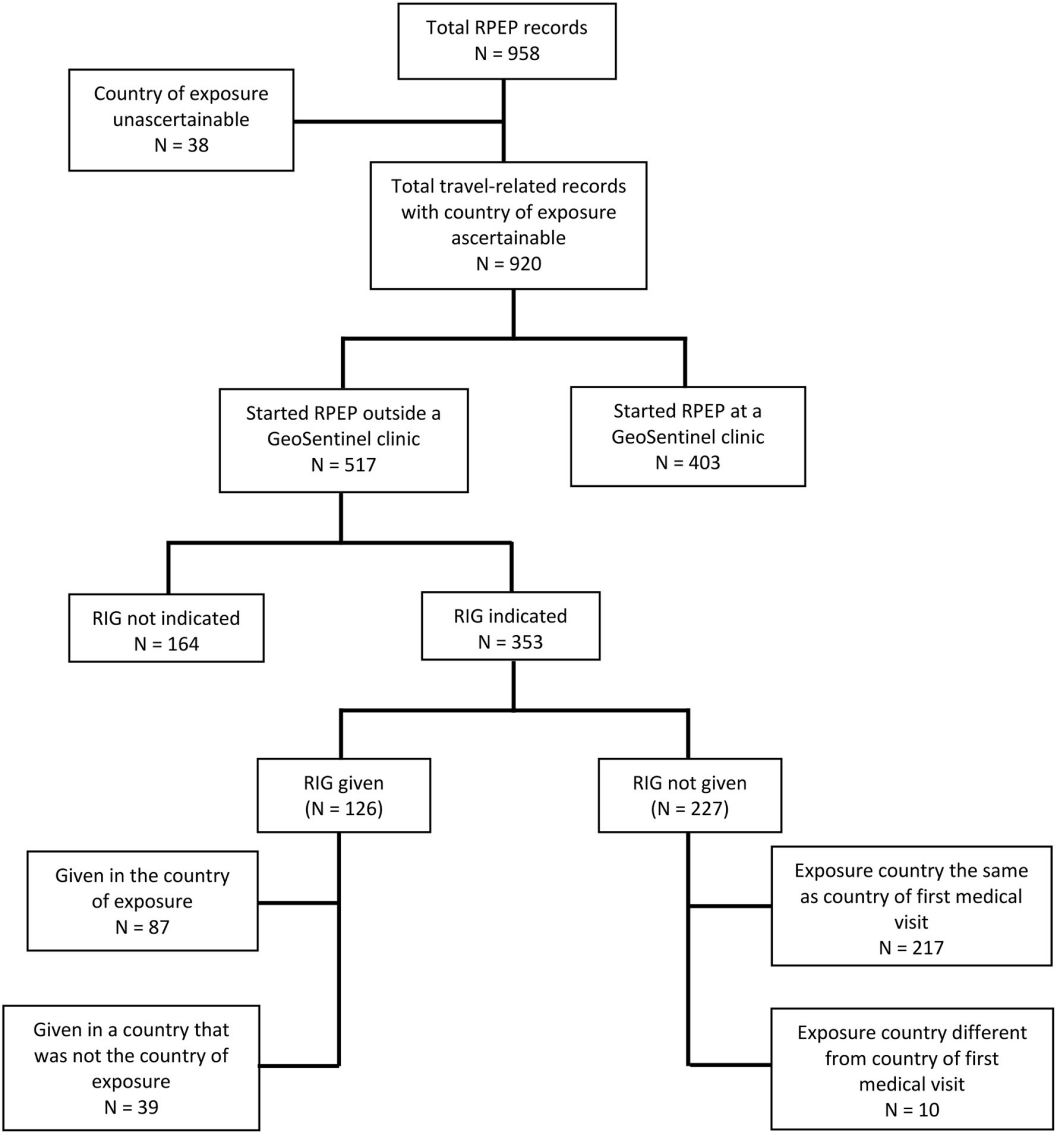
Number of international travelers receiving RPEP reported to GeoSentinel, September 2014–July 2017 (RIG: Rabies immunoglobulin; RPEP: Rabies post-exposure prophylaxis).

We examined 958 records; 38 were excluded (Fig 1). The analysis included 920 international travelers; 517 (56.2%) started RPEP before presenting to a GeoSentinel clinic (Group A), and 403 (43.8%) started RPEP at a GeoSentinel clinic (Group B). Travelers were assessed at 33 GeoSentinel clinics in 21 countries. Median age was 30 years (range 0–87), 52.3% were female, and 39.8% did not seek pre-travel advice (Table 2). The most frequent purpose of travel was tourism (731 travelers; 79.5%), followed by travelers visiting friends and relatives (103 travelers; 11.2%). Over 98% of travelers were seen as outpatients. Most travelers were exposed in Asia (697 travelers; 75.9%); 550 travelers (59.8%) were exposed in Southeast Asia; 115 travelers (12.5%) were exposed in South Central Asia; and 32 (3.5%) were exposed in North East Asia. Thailand, Indonesia, and Nepal accounted for over 50% of all exposures; the most frequent location of exposure was Bali, Indonesia [64 of 325 (19.7%) travelers with information available]. A greater proportion of travelers from Group A (versus Group B), were visiting friends and relatives (VFRs) (16.1% versus 6.0%) or were exposed in Southeast Asia (68.5% versus 48.6%). Detailed data will be made available on request.

**Table 2:** Demographics, travel characteristics, and clinical presentation of travelers reported to GeoSentinel who received rabies post-exposure prophylaxis, September 2014–July 2017 (N = 920).

	Travelers receiving an RPEP diagnosis (N = 920)	Travelers who started RPEP before presenting to a GeoSentinel clinic (N = 517) (Group A)	Travelers who did not start RPEP before presenting to a GeoSentinel clinic (N = 403) (Group B)
Median age (range) in years	30 (0–87)^1^	29 (1–87)^2^	31 (0–81)^3^
Female n (%)	481 (52.3)	266 (51.5)	215 (53.5)
Top three reasons for travel n (%)	Tourism: 731 (79.5) VFR: 103 (11.2) Business/corporate/conference: 44 (4.8)	Tourism: 401 (77.6) VFR: 83 (16.1) Business/corporate/conference: 20 (3.9)	Tourism: 330 (81.9) Business/corporate/conference: 24 (6.0) VFR: 20 (5.0)
Pre-travel encounter n (%)	Yes: 158 (17.2) No: 366 (39.8) Don’t know: 396 (43.0)	Yes: 79 (15.3) No: 189 (36.6) Don’t know: 249 (48.1)	Yes: 79 (19.6) No: 177 (43.9) Don’t know: 147 (36.5)
Top five regions of exposure n (%)	Southeast Asia: 550 (59.8) South Central Asia: 115 (12.5) North Africa: 66 (7.2) North East Asia: 32 (3.5) Sub-Saharan Africa: 30 (3.2)	Southeast Asia: 354 (68.5) South Central Asia: 44 (8.5) North Africa: 43 (8.3) North East Asia: 22 (4.3) Middle East: 15 (3.0)	Southeast Asia: 196 (48.6) South Central Asia: 71 (17.6) North Africa: 23 (5.7) Sub-Saharan Africa: 21 (5.2) South America: 18 (4.5)
Top five countries of exposure n (%)	Thailand: 271 (29.5) Indonesia: 142 (15.4) Nepal: 48 (5.2) Vietnam: 43 (4.7) Cambodia: 35 (3.8)	Thailand: 196 (37.9) Indonesia: 82 (16.1) Algeria: 23 (4.5) Cambodia: 23 (4.5) Vietnam: 23 (4.5)	Thailand: 75 (18.6) Indonesia: 60 (14.9) Nepal: 47 (11.7) Vietnam: 20 (5.0) Cambodia: 12 (3.0) India: 12 (3.0)

RPEP = rabies post-exposure prophylaxis, VFR = visiting friends and relatives

^1^Of 910 with information available

^2^Of 509 with information available

Nine (3%) travelers in Group A had received a complete 3-dose PrEP regimen; five received two-dose and five single-dose PrEP regimens. Most sought health care on the day of animal exposure (median 0 days; range 0–366 days; interquartile range: 0–1 days). Travelers were exposed most frequently to dogs (260; 50.3%), non-human primates (NHPs) (182; 35.2%), cats (59; 11.4%), or bats (4; 2.1%). Travelers to Thailand, Indonesia, and Cambodia were exposed more frequently to NHPs than to other animals, and tourists were exposed more frequently to NHPs than were VFRs (42.6% and 3.6%, respectively). Among Group A exposures, 362 (70.0%) were classified as WHO category III, 112 (21.7%) were category II, and 17 (3.3%) were category I [3]. Only 133 (25.7%) exposures occurred in rural areas, while 210 (40.6%) were in cities [the remaining 174 (33.7%) were unknown]. A total of 125 (24.2%) travelers reported being exposed through an unprovoked bite, and 112 (21.7%) reported visiting an animal reserve.

In Group A, 353 (68.3%) had an indication for RIG according to WHO criteria (Fig 1). Of these, 126 (35.7%) received RIG; 87 (24.7%) travelers received RIG in the country of exposure. The remaining 227 travelers (64.3%) did not receive RIG (Fig 1). Among the 227 travelers who did not receive RIG although indicated, 144 (63.4%) presented to a GeoSentinel clinic when RIG administration was no longer indicated (>7 days after the first dose of vaccine) [3]. Travelers exposed in Indonesia were less likely to receive RIG in the country of exposure (RR: 0.30; 95% CI: 0.12–0.73; P = 0.01) (Table 3).

**Table 3:** Relative risk of receiving rabies immunoglobulin in the country of exposure when indicated, by traveler and exposure characteristics, September 2014–July 2017 (N = 304).

Characteristic		RIG given in country of exposure (N = 87)	RIG not given in country of exposure (N = 217)	Relative risk of receiving RIG in the country of exposure (95% CI)	*P* value
Median age (range) in years		32 (1–87)^1^	30 (2–79)		
Female n (%)		45 (51.7)	101 (46.5)	1.11 (0.9–1.4)	0.40
Travel reason n (%)	Tourism	66 (75.9)	169 (77.9)	0.97 (0.8–1.1)	0.71
	VFR	17 (19.5)	35 (16.1)	1.21 (0.7–2.4)	0.47
	Business	3 (3.5)	7 (3.2)	1.07 (0.3–4.0)	0.92
	Missionary	0 (0)	2 (0.9)	0.50 (0.0–10.2)	0.65
	Education	1 (1.2)	2 (0.9)	1.25 (0.1–13.6)	0.86
	Migrant worker	0 (0)	1 (0.5)	0.83 (0.0–20.1)	0.91
	Migration	0 (0)	1 (0.5)	0.83 (0.0–20.1)	0.91
Pre-travel rabies immunization n (%)	None	83 (95.4)	200 (92.2)	1.04 (1.0–1.1)	0.26
	Unknown	2 (2.3)	9 (4.2)	0.55 (0.1–2.5)	0.44
	One dose	1 (1.2)	4 (1.8)	0.62 (0.1–5.5)	0.67
	Two doses	1 (1.2)	4 (1.8)	0.62 (0.1–5.5)	0.67
Countries of exposure n (%)	Thailand	43 (49.4)	78 (39.5)	1.38 (1.0–1.8)	0.02
	Indonesia	5 (5.8)	42 (19.4)	0.30 (0.1–0.7)	0.01
	India	3 (3.4)	13 (6.0)	0.58 (0.2–2.0)	0.38
	China	1 (1.2)	12 (5.5)	0.21 (0.0–1.6)	0.13
	Algeria	6 (6.9)	7 (3.2)	2.14 (0.7–6.2)	0.16
	Philippines	8 (9.2)	1 (0.5)	19.95 (2.5–157.2)	0.01
	Sri Lanka	8 (9.2)	5 (2.3)	3.99 (1.3–11.9)	0.013
Exposure location n (%)	City	35 (40.2)	90 (41.5)	0.97 (0.7–1.3)	0.84
	Unknown	32 (36.8)	72 (33.2)	1.11 (0.8–1.5)	0.55
	Rural	20 (23.0)	55 (25.4)	0.91 (0.6–1.4)	0.67
Exposure activity n (%)	Unknown	33 (37.9)	68 (31.3)	1.21 (0.9–1.7)	0.26
	Unprovoked bite	23 (26.4)	49 (22.6)	1.17 (0.8–1.8)	0.47
	Visiting animal park or reserve	11 (12.6)	46 (21.2)	0.60 (0.3–1.1)	0.10
	Walking	13 (14.9)	39 (18.0)	0.83 (0.5–1.5)	0.53
	Other	4 (4.6)	9 (4.2)	1.11 (0.4–3.5)	0.86
	Working with animals	1 (1.2)	6 (2.8)	0.42 (0.1–3.4)	0.41
	Biking	2 (2.3)	0 (0)	12.39 (0.6–255.4)	0.10
Animal exposure n (%)	Dog	53 (60.9)	109 (50.2)	1.21 (1.0–1.5)	0.08
	Non-human primate	22 (25.3)	75 (34.5)	0.73 (0.5–1.1)	0.13
	Cat	9 (10.3)	26 (12.0)	0.86 (0.4–1.8)	0.69
	Other	2 (2.3)	6 (2.8)	0.83 (0.2–4.0)	0.82
	Bat	1 (1.2)	1 (0.5)	2.47 (0.2–39.0)	0.52

RIG = rabies immunoglobulin, CI = confidence interval, VFR = visiting friends and relatives

^1^Of 86 travelers for whom information was available

Travelers exposed in Thailand (RR 1.38, 95% CI: 1.0–1.8; P = 0.02], Sri Lanka (RR 3.99, 95% CI: 3.99–11.9; P = 0.013), and the Philippines (RR 19.95, 95% CI: 2.5–157.2; P = 0.01), were more likely to receive RIG in the country of exposure. There were no significant differences in demographic, travel, or exposure characteristics between those who received RIG and those who did not. None of the travelers included in this analysis were known to have developed rabies.

## Discussion

This analysis supports the findings of previous studies that reported rabies exposures among international travelers occur most frequently in Asia and that tourists sustain more rabies exposures than other types of travelers [2]. Of major concern is the finding that almost 65% of travelers in Group A with an indication for RIG did not receive it. This finding is likely multifactorial and may be due to limited availability of RIG at both the national and primary care levels of the health system, which may be due to procurement difficulties, the need to store RIG at 2–8°C [14], and its high cost. In addition, there may be insufficient awareness of indications for the use of RIG. Information about RIG availability at travel destinations can be difficult to find, and supply may be inconsistent. One survey, although biased toward international travel medicine organizations, demonstrated that less than half of clinics surveyed in Asia had access to RIG [15]. Similarly, a survey of US Embassy medical staff who provided health advice, conducted by the same group of investigators, found that possible rabies exposures accounted for about 2% of health inquiries. About two-thirds of the respondents in the latter survey reported that RIG was available for travelers in the country where they were based. Notably in Southeast and East Asia, human RIG was often not available [16]. In our analysis, Bali, Indonesia, was the most common location to have an exposure to a potentially rabid animal, but very few travelers received RIG in Indonesia when indicated. Since more than 90% of travelers were not completely vaccinated before traveling, and over 70% of travelers sustained a category III exposure, it is imperative to identify high-risk areas where RIG may not be widely available, such as Bali, so clinicians can encourage PrEP for travelers to these destinations and educate travelers about steps to take if they sustain animal bites.

Following the Strategic Advisory Group of Experts on immunization (SAGE) meeting in October 2017, WHO updated its recommendation for PrEP to two doses (days 0 and 7) instead of three for immunocompetent individuals [17]. The rationale for this decision is that several studies have demonstrated similar immunogenicity after one week compared to the classical 3–4-week regimens [17]. Reducing the time frame and number of doses required for PrEP would make it simpler and more cost-effective to implement in travelers as the classic 3-dose PrEP may be difficult to complete with the short average interval to departure (<21 days) of many travelers [18,19]. High vaccine cost is another reason for very low PrEP coverage in travelers [20]. The new WHO recommendations will hopefully allow increasing rabies pre-travel vaccination coverage in travelers to destinations where RIG administration is unlikely to be provided despite being indicated. Although a single dose of PrEP should confer some protection, in the event of a potential rabies exposure, WHO recommends full RPEP including RIG if indicated [17].

Given the travel and tropical medicine specialization of GeoSentinel sites, these data may not be representative of all international travelers who receive RPEP or of travelers to a particular country or region. GeoSentinel data are not generalizable, so risk estimates of a particular illness cannot be calculated. Additional information (i.e., urban or rural exposure, activity during exposure, type of animal exposure, and pre-travel rabies immunization receipt) was collected only on those travelers in Group A; data on whether RIG was provided or offered at GeoSentinel clinic visits were not collected. Information collected from travelers who started RPEP outside a GeoSentinel clinic was based upon patient report and clinical description of the WHO exposure category and is subject to observer and recall bias. We did not collect data on reasons for delay in treatment. Despite these limitations, this analysis identifies specific countries where travelers may be more or less likely to receive RIG and helps inform education of international travelers regarding appropriate rabies prevention.

Travelers should seek pre-travel advice before traveling abroad to ensure they receive proper education on avoiding contact with animals and what to do after an animal exposure, notably regarding indications for RIG. Since rabies exposures are often unpredictable, travelers must be reminded that exposures often come from animals they perceive as unthreatening (e.g., NHPs at tourist locations). When performing a risk assessment at a pre-travel consultation, health-care practitioners must assess if travelers’ destinations are at high risk for rabies (and/or with unavailable RIG) and the possibility for additional or repeated exposures when considering offering PrEP [21].
